# In Vitro Antioxidant Activity and Dual-Target Inhibitory Effects of Dipyridamole on α-Amylase and α-Glucosidase: Molecular Mechanisms and Molecular Dynamics Simulations

**DOI:** 10.3390/biom16091361

**Published:** 2026-09-18

**Authors:** Cong Zhao, Hongfei Liu, Yuting Shu, Jingchen Wei, Ruiqian Feng, Lina Chen, Helong Bai, Jing Wang, Dongfang Shi

**Affiliations:** 1 College of Life Sciences, Changchun Normal University, Changchun 130032, China; zhaocong@ccsfu.edu.cn; 2 College of Chemistry, Changchun Normal University, Changchun 130032, China; 3 Institute of Science and Technology Innovation, Changchun Normal University, Changchun 130032, China

**Keywords:** dipyridamole, antioxidant, α-amylase, α-glucosidase

## Abstract

α-Amylase and α-glucosidase are complementary therapeutic targets in type 2 diabetes; simultaneous inhibition provides a dual-blockade strategy to suppress postprandial hyperglycemia. Dipyridamole, a conventional antiplatelet drug with proven anti-inflammatory, antioxidant, and antiproliferative effects and a favorable safety profile, is a candidate for repurposing. This study assessed in vitro antioxidant activity and inhibitory actions against both enzymes, and employed molecular docking, molecular dynamics simulations to elucidate mechanisms. Dipyridamole showed EC_50_ values of 589.7 μM (ABTS+·), 652.2 μM (·OH), and 1607.1 μM (DPPH·), and IC_50_ values of 257.6 μM (α-amylase) and 348.8 μM (α-glucosidase). Molecular docking yielded binding free energies of −6.5 and −6.0 kcal/mol for α-amylase and α-glucosidase, respectively, with binding mediated mainly by hydrogen bonds and hydrophobic interactions. Molecular dynamics simulations revealed that, under the present simulation conditions, both the apo forms of α-amylase and α-glucosidase and their respective dipyridamole complexes exhibited relatively stable dynamical behavior throughout the course of the trajectories. Overall, dipyridamole showed not only moderate free radical scavenging capacity but also inhibitory activity against α-amylase and α-glucosidase, suggesting that this molecule may serve as a promising lead compound for further relevant drug development and functional exploration.

## 1. Introduction

According to a report released by the International Diabetes Federation (IDF) in 2024, the global prevalence of diabetes has reached 11.11%, with the total number of patients exceeding 589 million. It is projected that by 2050, the number of people with diabetes worldwide will rise to 853 million, with the prevalence increasing to 12.96%, type 2 diabetes mellitus (T2DM) constitutes the most prevalent subtype of diabetes, representing 90–95% of all diagnosed cases worldwide [1]. Chronic blood glucose instability can damage various tissues and organs, leading to the development of multiple complications. α-Amylase and α-glucosidase are two critical rate-limiting enzymes in the process of carbohydrate digestion. α-Amylase primarily acts on α-1,4 glycosidic bonds in polysaccharides such as starch, breaking them down into smaller oligosaccharides including maltose, maltotriose, and dextrins [2]. Subsequently, α-glucosidase further hydrolyzes these oligosaccharides into glucose, which can be absorbed by the intestinal epithelium [3]. Therefore, inhibiting the activities of these two enzymes can effectively slow down the rate of carbohydrate breakdown, delays glucose release and absorption, and thereby prevent sharp postprandial blood glucose spikes. This strategy not only represents a core mechanism of pharmacological intervention in diabetes dietary management but also provides an important target for the development of natural hypoglycemic functional foods. On the other hand, the onset and progression of diabetes are closely associated with the state of oxidative stress in the body. Chronic hyperglycemia induces excessive production of reactive oxygen species (ROS) and reactive nitrogen species (RNS) through pathways such as glucose auto-oxidation, non-enzymatic protein glycation, and polyol pathway activation, leading to an imbalance in the oxidative–antioxidant system [4]. Excess free radicals can damage pancreatic β-cells, reduce their insulin synthesis and secretion capacity, while also exacerbate peripheral insulin resistance [5]. Therefore, scavenging free radicals and enhancing endogenous antioxidant enzyme activity can help alleviate the toxic effects of oxidative stress on β-cells, protect and improve islet function, and thus play a positive role in the prevention and treatment of diabetes and its complications. In summary, simultaneously targeting carbohydrate-digesting enzymes and oxidative stress pathways has become an effective comprehensive strategy for controlling postprandial blood glucose and delaying the progression of diabetes.

Dipyridamole, an antiplatelet agent that has undergone decades of clinical validation, is widely used for the secondary prevention of cardiovascular and cerebrovascular diseases. Its classic mechanisms of action primarily include the inhibition of phosphodiesterase activity and the blockade of adenosine reuptake, leading to elevated intracellular cyclic adenosine monophosphate (cAMP) levels in platelets and a synergistic inhibition of platelet aggregation [6]. In recent years, with the deepening of drug repurposing research, the pleiotropic pharmacological activities of dipyridamole have been gradually revealed, and its potential in the field of metabolic regulation has attracted particular attention. It has been demonstrated that dipyridamole possesses significant antioxidant properties, effectively inhibiting the generation of ROS in platelets and vascular endothelial cells [7], thereby alleviating oxidative stress injury—a mechanism that is of positive significance for delaying the progression of atherosclerosis and protecting vascular function [8]. More importantly, dipyridamole has also been shown to inhibit α-amylase and α-glucosidase, two key carbohydrate-digesting enzymes [9]. Inhibition of their activities delays the breakdown of complex carbohydrates and glucose absorption in the intestine, thereby reducing postprandial blood glucose fluctuations. Therefore, dipyridamole exhibits a dual nature, combining antioxidant and glucosidase-inhibitory activities, which confers a unique “drug repurposing” prospect for its intervention in diabetes mellitus and its complications. For patients at high risk of cardiovascular and cerebrovascular diseases, as well as those with impaired glucose tolerance or early-stage diabetes, dipyridamole, or its structurally optimized derivatives, may offer a “two-in-one” therapeutic benefit. At present, research on the metabolic regulatory effects of dipyridamole is still in the exploratory stage, both in vitro and in vivo. Nevertheless, it has already pointed to a new direction for drug function re-evaluation and indication expansion, and it is anticipated that systematic preclinical and clinical studies will further validate its real value in the comprehensive management of metabolic diseases in the future.

In this study, the in vitro antioxidant activity of dipyridamole and its inhibitory effects on α-amylase and α-glucosidase were systematically evaluated. Furthermore, its molecular mechanisms were elucidated using molecular docking, and molecular dynamics simulations, thereby providing a basis for subsequent drug development and functional exploration.

## 2. Materials and Methods

### 2.1. Reagents and Materials

Dipyridamole, L-ascorbic acid (Vc), ferrous sulfate, 2,2-Diphenyl-1-Picrylhydrazyl (DPPH), hydrogen peroxide, salicylic acid, 2,2′-Azinobis-(3-Ethylbenzthiazoline-6-Sulphonic acid) (ABTS), Acarbose (ACB), α-amylase (from *Aspergillus oryzae*, EC 3.2.1.1) and α-glucosidase (from *Bacillus stearothermophilus*, EC 3.2.1.20) were purchased from Shanghai Yuanye Biotechnology Co., Ltd. (Shanghai, China). All the other chemical reagents were analytically pure.

### 2.2. Determination of Antioxidant Activity

#### 2.2.1. ABTS+· Scavenging Assay

ABTS+· scavenging capacity assay was performed with minor modifications in reference to the method of Elsayed [10]. To prepare the ABTS+· stock solution, 7 mM ABTS diammonium salt solution was mixed with a small volume of 140 mM potassium persulfate (K_2_S_2_O_8_) stock solution [11]. This mixture was then incubated in the dark at 4 °C for 12 h, to facilitate the generation of ABTS+·. Subsequently, 100 µL of dipyridamole samples with various concentrations (0.1–1.0 mg/mL; 0.198–1.98 mM) were separately mixed thoroughly with 100 µL of the ABTS+· stock solution. After standing in the dark for 30 min, the absorbance of each reaction mixture was measured at a wavelength of 734 nm. Each group of experiments was performed in triplicate to ensure the reliability and reproducibility. The absorbance of the sample reaction system was denoted as Ai, while A0 represented the absorbance measured by substituting the sample with the sample solvent. Aj was defined as the absorbance obtained when the ABTS+· solution was replaced by the sample solvent. Vc served as the positive control, and the ABTS+· radical scavenging activity was calculated by applying the following Formula (1):

ABTS+· scavenging rate = (1− (AI − Aj)/A0) × 100%(1)

#### 2.2.2. ·OH Scavenging Assay

·OH was determined using the method of Kotora with minor modifications [12]. A volume of 50 µL dipyridamole sample solutions (serial concentrations: 0.1–1.0 mg/mL; 0.198–1.98 mM) was mixed with 50 µL of 50 mmol/L ferrous sulfate (FeSO_4_) solution, 50 µL of 10 mmol/L salicylic acid–ethanol solution, and 50 µL of 3% hydrogen peroxide (H_2_O_2_) solution. The resulting mixture was incubated in the dark for 30 min. Triplicate measurements were carried out for each experimental group to ensure the reliability and repeatability of the results. After the incubation period, the absorbance of reaction system (designated as A_reaction_) was measured at a wavelength of 510 nm. For the control group (A_control_), distilled water was used to replace the salicylic acid–ethanol solution, and the corresponding absorbance was recorded. For the blank group (A_blank_), the sample was substituted with the sample solvent, and its absorbance was measured as well. Vc was employed as the positive control, and the ·OH scavenging capacity was computed in accordance with the following Formula (2):

·OH scavenging rate = (1 − (A_reaction_ − A_control_)/A_blank_) × 100% (2)

where A_reaction_ represents the absorbance of the sample, A_control_ represents the absorbance of the blank control group, and A_blank_ represents the absorbance of the sample without hydrogen peroxide.

#### 2.2.3. DPPH· Scavenging Assay

The experiments were carried out using the method described in Kotora [12]. Dipyridamole samples with different concentration gradients (100 µL each, serial concentrations: 0.1–1.0 mg/mL; 0.198–1.98 mM) were individually mixed with 100 µL of DPPH· solution. Each experimental group was run in triplicate. After incubating the mixture for 30 min, the absorbance (denoted as Ai) was determined at a wavelength of 517 nm. The control absorbance values were recorded: Aj was measured by substituting the dipyridamole sample with the sample solvent, while Ac was obtained by replacing the DPPH· solution with anhydrous ethanol. Vc was used as the positive control, and the DPPH· scavenging capacity was calculated by means of the following Formula (3):

DPPH· scavenging rate = (1 − (Ai − Aj)/Ac) × 100%(3)

### 2.3. Enzyme Inhibition Assay

#### 2.3.1. Determination of α-Amylase Inhibition Rate

The experiments were carried out using the method described in Padilla-Camberos [13], with minor modifications. The enzyme working solution was freshly prepared in phosphate-buffered saline (PBS, 10 mM, pH 6.9) prior to use and stored at −20 °C when not in immediate use. The substrate, soluble starch, was prepared as a 1% (*w*/*v*) solution in PBS. Dipyridamole stock solution (2.5 mg/mL) was prepared in PBS with the aid of dimethyl sulfoxide (DMSO) as a co-solvent, and subsequently diluted with PBS to achieve the other reagent solutions, the final concentration of DMSO in all reaction mixtures was maintained at ≤5% (*v*/*v*) to ensure solubility without affecting enzyme activity, as previously reported [14,15]. In a typical assay, 100 µL of dipyridamole solution at various concentrations was mixed with 50 µL of α-amylase (1.0 U/mL). The mixture was pre-incubated at 37 °C for 15 min. Then, 50 µL of 1% (*w*/*v*) soluble starch was added to initiate the reaction, and the mixture was incubated at 37 °C for an additional 10 min. The reaction was terminated by adding 100 µL of 3,5-dinitrosalicylic acid (DNS) reagent, followed by heating in a boiling water bath for 5 min. After cooling to room temperature, the reaction mixture was diluted with 1 mL of distilled water, and the absorbance was measured at 540 nm using a microplate reader.

The final concentrations of dipyridamole and acarbose in the 1.3 mL total reaction system was 0.038, 0.077, 0.115, 0.154 and 0.192 mg/mL, respectively. All experiments were performed in triplicate. ACB was used as a positive control. The inhibition rate was calculated using the following Equation (4):

Inhibition rate = (1 − (A1 − A2)/(A0 − A0′)) × 100%(4)

where A1, A2, A0, and A0′ are the absorbance at 540 nm for the dipyridamole sample group, blank sample group, control group (without inhibitor, containing equivalent DMSO), and blank control group (without enzyme), respectively. IC_50_ was determined from the regression equation of the inhibition curve.

#### 2.3.2. Determination of α-Glucosidase Inhibition Rate

The experiments were carried out using the method described in Yao [16], with minor modifications. The enzyme working solution was freshly prepared in PBS (10 mM, pH 6.9) prior to use and stored at −20 °C when not in immediate use. The substrate, PNPG was prepared as a 5.0 mmol/L solution in PBS. The preparation of dipyridamole solution is the same as in Section 2.3.1. In a typical assay, 100 µL of dipyridamole solution at various concentrations was mixed with 50 µL of α-glucosidase (0.5 U/mL). The mixture was pre-incubated at 37 °C for 15 min. Then, 100 µL of 5.0 mmol/L PNPG was added to initiate the reaction, and the mixture was incubated at 37 °C for an additional 10 min. The reaction was terminated by adding 750 µL of 1.0 mol/L Na_2_CO_3_ solution. After thorough mixing, the absorbance was measured at 405 nm using a microplate reader.

The final concentrations of dipyridamole and acarbose in the 1.0 mL total reaction system was 0.05, 0.1, 0.15, 0.2 and 0.25 mg/mL, respectively. All experiments were performed in triplicate. Acarbose was used as a positive control. The inhibition rate was calculated using the following Equation (5):

Inhibition rate = (1 − (A1 − A2)/(A0 − A0′)) × 100%(5)

where A1, A2, A0, and A0′ are the absorbance at 405 nm for the sample group, blank sample group, control group (without inhibitor, containing equivalent DMSO), and blank control group (without enzyme), respectively. IC50 was determined from the regression equation of the inhibition curve.

### 2.4. Molecular Docking

The two-dimensional (2D) structures of the small-molecule ligands were retrieved from the PubChem database, with the target proteins being α-amylase (PDB ID: 1XH0) and α-glucosidase (PDB ID: 2QMJ). The ligands were imported into Chem3D 23.1.1 software for three-dimensional (3D) structure construction and subsequent energy minimization, and were finally saved in mol2 format. The crystal structures of the protein targets were obtained from the RCSB Protein Data Bank (PDB) by selecting high-resolution structures [17]. Prior to docking, the protein structures were pre-processed using PyMOL 2.6 software to remove water molecules, phosphate ions, and other non-essential groups, and were subsequently saved in PDB format. Molecular docking preparations, including hydrogen atom addition, removal of crystallographic water, assignment of partial charges, and definition of rotatable bonds for both proteins and ligands, were carried out using AutoDock tools (version 4) [18]. Docking simulations were performed with AutoDock Vina 1.2.5 [19]. To justify the binding-site preference and selectivity, a blind docking procedure was first performed over the entire surface of each enzyme, with the grid box covering the full protein structure without any positional bias toward the known active pocket. The top-ranked binding poses from the blind docking were found to be clustered predominantly within the known active-site pockets, confirming that the ligands preferentially bind to the enzyme active sites rather than to random surface regions. Subsequently, focused docking was carried out using a cubic docking grid box of 25 Å × 25 Å × 25 Å defined for both receptors. The grid box center coordinates were set to (7.252, 15.215, 40.065) for α-amylase and (−22.172, −6.329, −4.233) for α-glucosidase, based on the respective active-site geometries identified from the blind docking results. Each receptor was individually docked with the three ligands, namely Acarbose, Dipyridamole, and an additional test ligand (designated as lig), and the corresponding output files were generated in pdbqt format. The most favorable binding conformations were selected based on the binding free energy scores calculated by the docking program. The resulting protein–ligand interactions were visualised and analysed using Discovery Studio 2019 and PyMOL 2.6 software, from which two-dimensional interaction diagrams and three-dimensional binding mode illustrations were generated.

### 2.5. Molecular Dynamics Simulation

Molecular dynamics simulations of the protein–ligand complexes were performed using GROMACS 2022.2 [20]. The protein PDB file was converted into GROMACS-compatible topology and coordinate files using the pdb2gmx tool in GROMACS 2022.2, based on the selected Amber99SB-ILDN force field [21]. Ligand topology parameters based on the GAFF2 force field [22] were generated using the sobtop_1.0 (dev3.1) program. RESP charges for the ligand were derived from quantum-chemical calculations using Gaussian 16 software: geometry optimization and single-point energy calculations were performed at the B3LYP/6-31G(d) level, followed by RESP charge fitting using Multiwfn. The GAFF2 ligand parameters were verified and no unreasonable bond length or bond angle parameters were found. The systems were solvated using the TIP3P water model in a cubic simulation box, with a minimum distance of 1.0 nm between the protein atoms and the box edges to ensure adequate solvation. To mimic the physiological environment and maintain overall electroneutrality, Na^+^ and Cl^−^ ions were added using the gmx genion tool to a final concentration of 0.15 M NaCl. Long-range electrostatic interactions were treated using the Particle Mesh Ewald (PME) method, and the cutoff radius for both van der Waals and short-range electrostatic interactions was set to 1.2 nm. All bonds were constrained using the LINCS algorithm, and the integration time step was set to 2 fs. Prior to the production simulations, energy minimization was performed sequentially using the steepest descent and conjugate gradient methods to eliminate unreasonable steric clashes and locally high-energy conformations. Following energy minimization, a 1 ns NVT equilibration and a 1 ns NPT equilibration were carried out. The NVT equilibration was performed using the v-rescale thermostat at 300 K with a temperature coupling time constant of 1 ps. During the NPT equilibration, the pressure was maintained at 1 bar using the c-rescale barostat with a pressure coupling time constant of 1 ps. After the systems were sufficiently equilibrated, unrestrained molecular dynamics simulations were conducted. For each system, three independent 100 ns production MD runs were performed, each with a different random seed for initial velocity generation. Following the simulations, subsequent data analyses were carried out using the analysis tools bundled with GROMACS 2022.2: root-mean-square deviation (RMSD) of the protein backbone was calculated using gmx rms to assess overall structural stability; root-mean-square fluctuation (RMSF) of protein residues was calculated using gmx rmsf to evaluate local flexibility; the radius of gyration (Rg) was calculated using gmx gyrate to characterize the overall compactness of the protein; hydrogen bonds between the protein and the ligand were counted using gmx hbond to assess intermolecular interaction stability; and solvent-accessible surface area (SASA) was calculated using gmx sasa to analyze protein–solvent interactions. Binding free energies of the complexes were calculated using the MM-PBSA method via gmx_mmpbsa, to further evaluate the stability, structural changes, and solvent effects of the systems.

### 2.6. Statistical Analysis

All experiments were performed in three independent replicates, with three parallel samples in each replicate to obtain mean values. Data are expressed as mean ± standard deviation (SD). Statistical analyses were carried out using GraphPad Prism 10 and Origin 2024 software. One-way analysis of variance (ANOVA) followed by multiple comparison tests was applied for data evaluation, and a *p*-value of less than 0.05 was considered statistically significant.

## 3. Results and Discussion

### 3.1. Antioxidant Activity of Dipyridamole

**Figure 1:**
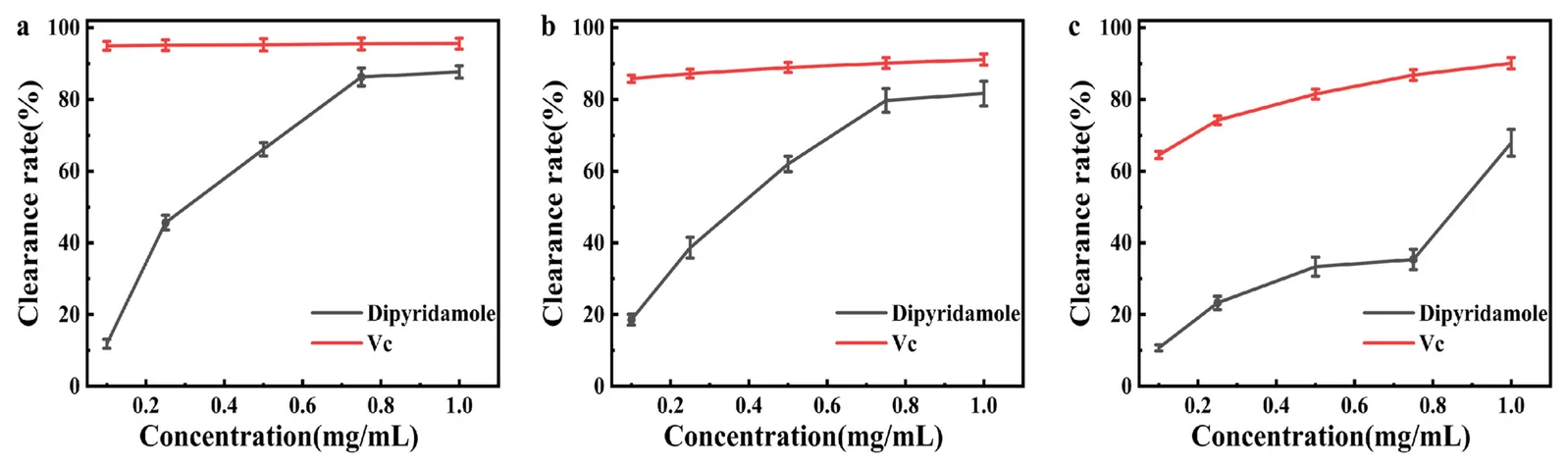
Scavenging rates of dipyridamole against three free radicals: (**a**) ABTS+· radical; (**b**) ·OH radical; (**c**) DPPH· radical.

Oxidative stress due to aberrant metabolism is considered as a crucial contributor to diabetes and its complications [23]. In vitro antioxidant assays serve as essential high-throughput tools for the initial identification of bioactive compounds, allowing for the establishment of structure–activity relationships that guide the rational design of more antioxidant agents [24]. However, these chemical-based assays do not account for physiological factors such as bioavailability and metabolism, and therefore should be complemented by cell-based models or in vivo studies to more reliably predict the biological efficacy of candidate compounds [25]. The redox properties of dipyridamole have been documented previously, with its antioxidant activity attributed to the reversible oxidation of phenolic hydroxyl groups to quinone structures [7]. The present study extends these observations by comparing the effects of dipyridamole against distinct radical species. In this study, the scavenging activities of dipyridamole against ABTS+·, ·OH, and DPPH· were evaluated, with Vc used as a positive control [26]. Dipyridamole exhibited concentration-dependent scavenging activity against all three radicals, with EC_50_ values of 0.2976 mg/mL (589.7 μM), 0.3291 mg/mL (652.2 μM), and 0.8110 mg/mL (1607.1 μM) for ABTS+·, ·OH, and DPPH·, respectively (Figure 1). These results indicated that dipyridamole possessed broad-spectrum free radical scavenging ability, with the better efficacy against ABTS+·, likely attributable to the electron-rich moieties (e.g., nitrogen-containing heterocycles) in its molecular structure that facilitate stabilization of cationic radicals [9]. Given that oxidative stress is a central pathological mechanism in metabolic diseases and their complications, the antioxidant property of dipyridamole may help alleviate tissue damage induced by oxidative stress. Compared with classical antioxidants (e.g., Vitamin C, Vitamin E) or certain flavonoids [27,28], the half-maximal effective concentrations of dipyridamole are within a comparable micromolar range. These findings suggest that dipyridamole may confer protection against oxidative stress, which warrants further investigation in cellular models. However, its distinct advantage lies in its well-established clinical safety profile, providing a convenient route for “drug repurposing” to counteract oxidative stress.

### 3.2. Inhibitory Activity of Dipyridamole Against α-Amylase and α-Glucosidase

**Figure 2:**
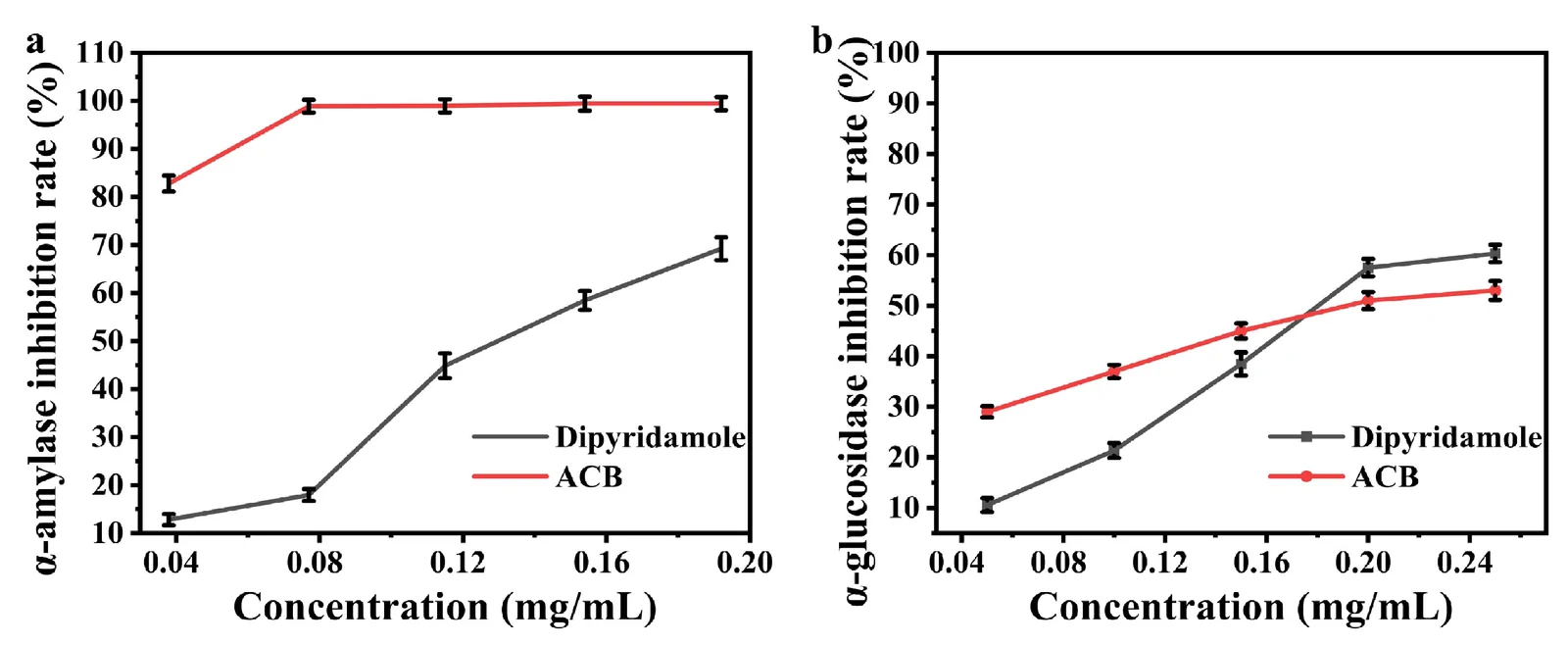
Inhibition rates of dipyridamole against α-amylase (**a**) and α-glucosidase (**b**).

Inhibiting the activities of α-amylase and α-glucosidase can directly suppress postprandial blood glucose elevation [29]. The inhibitory activities of dipyridamole against α-amylase and α-glucosidase increased in a concentration-dependent manner, the inhibition activity of α-amylase (IC_50_ of 0.130 mg/mL, 257.6 μM) is lower than that of ACB (IC_50_ of approximately 0.023 mg/mL, 35.6 μM), showed as Figure 2a, suggesting that dipyridamole is not suitable as a potent α-amylase inhibitor. This characteristic may actually offer a clinical advantage, as excessive α-amylase inhibition leads to undigested carbohydrates entering the colon, causing common adverse effects such as flatulence and diarrhea [30].

In contrast, dipyridamole displayed concentration-dependent enhancement of α-glucosidase inhibitory activity, dipyridamole showed inhibitory activity against α-glucosidase comparable to that of ACB, with IC_50_ values of 0.176 mg/mL (349 μM) and 0.190 mg/mL (294 μM), respectively (Figure 2b). α-Glucosidase is a key target for controlling postprandial blood glucose. Based on these in vitro enzyme-inhibition results, dipyridamole exhibits certain inhibitory potency toward this target, and may serve as a research molecule for exploring the delay of carbohydrate absorption. It should be noted that the in vitro assays employed *Aspergillus oryzae* α-amylase and *Bacillus stearothermophilus* α-glucosidase, both of microbial origin, rather than human enzymes. Given the structural differences between microbial and human homologs, caution is warranted in extrapolating these results, and future studies using human recombinant enzymes are needed.

It is worth noting that the inhibitory activities of dipyridamole measured in the present study (IC_50_ = 257.6 µM against α-amylase and 349 µM against α-glucosidase) are approximately 8.6- and 18-fold higher than those reported by Esmaeili et al. in mammalian enzyme systems (30.1 µM and 19.4 µM, respectively) [9]. This discrepancy is quantitatively consistent with structural differences in the active sites between species. Mammalian enzymes possess narrower hydrophobic pockets and are enriched with tyrosine/tryptophan aromatic residue clusters [31], which facilitate multivalent π–π stacking and hydrogen-bonding interactions with dipyridamole [32,33,34]. In contrast, the corresponding residues in microbial enzymes are often replaced by small non-polar amino acids [35], resulting in substantially reduced binding energies [36]. Furthermore, the IC_50_ value of ACB, the positive control used in this study, against α-glucosidase (294 µM) was also higher than that reported in the rat enzyme system (11.9 µM), suggesting that the two assay systems differ in overall sensitivity rather than reflecting a contradiction in dipyridamole’s intrinsic activity. Therefore, the weak inhibitory potency of dipyridamole observed with microbial enzymes should not be directly extrapolated to its pharmacological potential against mammalian (including human) orthologs. Nevertheless, the activity profile obtained in this study still provides useful information: dipyridamole exhibited inhibitory activity against α-glucosidase comparable to that of ACB, implying a possible capacity to delay carbohydrate absorption. Further investigations employing human recombinant enzymes or mammalian tissue-derived enzyme preparations, combined with in vivo starch loading tests, are warranted to comprehensively evaluate its glucose-lowering potential.

### 3.3. Molecular Docking Results

**Figure 3:**
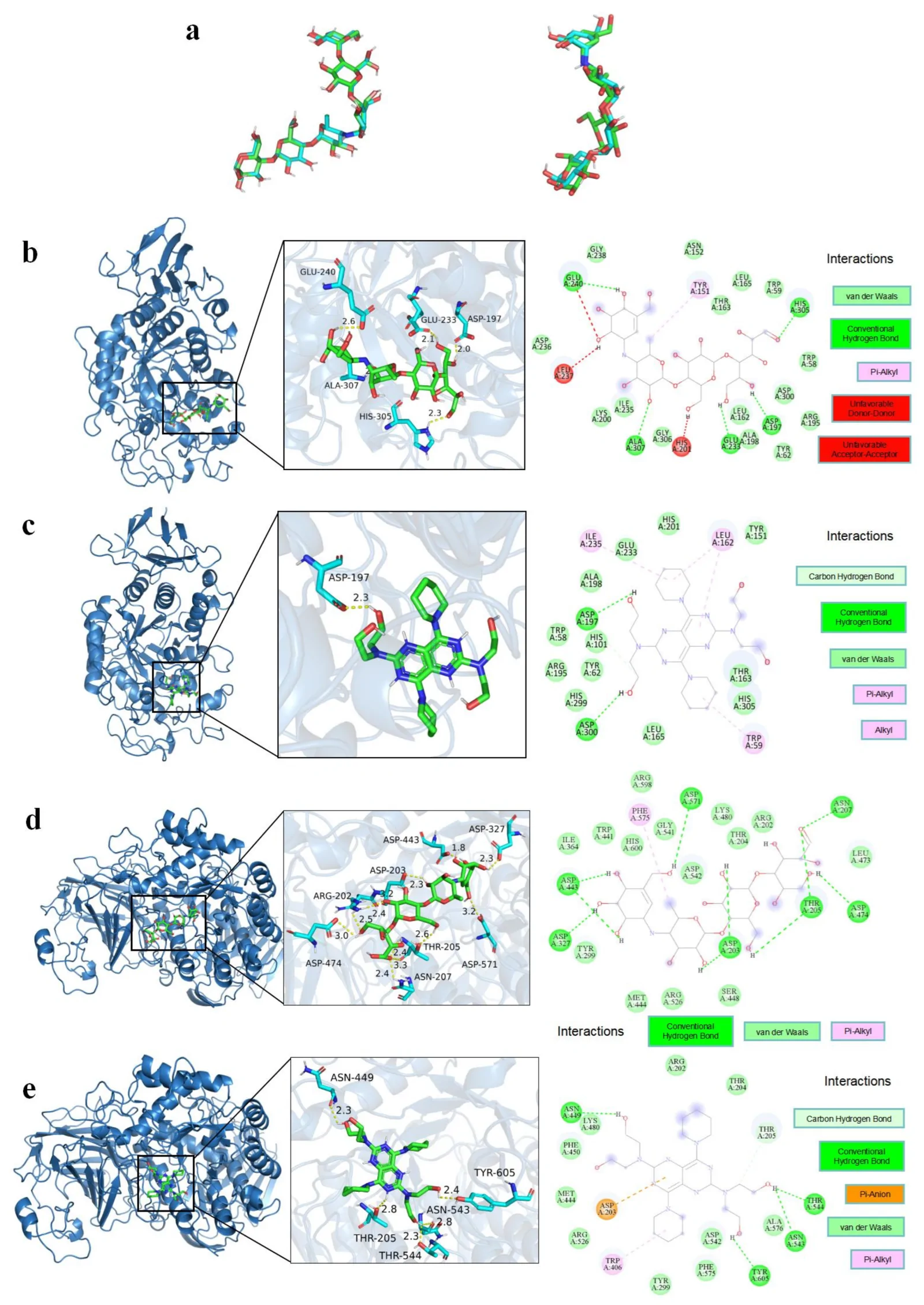
Molecular docking models of dipyridamole with enzymes: (**a**) Ligands of the α-amylase (**left**) and α-glucosidase (**right**) systems in re-docking validation. (**b**) Binding mode of acarbose to α-amylase as revealed by molecular docking. (**c**) Binding mode of dipyridamole to α-amylase as revealed by molecular docking. (**d**) Binding mode of acarbose to α-glucosidase as revealed by molecular docking. (**e**) Binding mode of dipyridamole to α-glucosidase as revealed by molecular docking.

Molecular docking was used to predict the binding sites, key amino acid residues, and binding forces between the small molecule and the macromolecules [37]. To evaluate the reproducibility of the docking procedure, the co-crystallized ligands were extracted from the native binding pockets of α-amylase and α-glucosidase and re-docked into their respective target proteins. The root-mean-square deviation (RMSD) values between the re-docked poses and the crystallographic ally determined conformations were calculated as 0.267 Å for α-amylase and 1.281 Å for α-glucosidase (Figure 3a). Both values fell well below the accepted threshold of 2.0 Å, confirming that the grid parameters and scoring functions employed were capable of accurately reproducing the native binding geometries.

The binding free energy of dipyridamole with α-amylase was calculated to be –6.5 kcal/mol, which is lower than the threshold typically required for active compounds, indicating that a stable complex can theoretically be formed between dipyridamole and α-amylase. In the docked conformation, the rigid, coplanar aromatic rings of dipyridamole engaged in hydrogen-bonding interactions exclusively with ASP197 and ASP300, with interatomic distances consistent with the canonical range for such interactions, thereby indicating a favorable geometric complementarity between the ligand and the binding cavity. The hydrophobic subpacket of α-amylase accommodated the aromatic moieties through contacts involving ILE235, LEU162, and TRP59. The surrounding van der Waals contact network comprised ten residues, namely TYR151, HIS201, GLU233, ALA198, TRP58, ARG195, HIS299, LEU165, HIS305, and THR163 (Figure 3c). Collectively, these observations suggest that dipyridamole exhibits a favorable binding affinity toward α-amylase.

The binding free energy of dipyridamole with α-glucosidase was determined to be –6.0 kcal/mol, which is lower than the binding energy threshold of most small molecule inhibitors, indicating the formation of a thermodynamically stable complex and good potential as an inhibitor. In the predicted binding mode, the pyrimidine-fused polycyclic core of dipyridamole participated in hydrogen-bonding interactions, including ASN449, TYR605, ASN543, and THR544. Additional polar stabilization is contributed by a Pi-Anion interaction with ASP203 and a Carbon Hydrogen Bond with THR205. Hydrophobic stabilization is achieved through contact with TRP406, while the surrounding van der Waals contact surface involves THR204, ARG202, LYS480, PHE450, MET444, ARG526, TYR299, PHE575, ASP542, and ALA576 (Figure 3e). Taken together, these findings indicate that dipyridamole occupies the active site of α-glucosidase in a stable manner, and the docking data offer a theoretical foundation for its prospective inhibitory activity against this enzyme.

Molecular docking results showed that the positive control drug ACB exhibited binding free energies of −6.7 kcal/mol with α-amylase and −6.6 kcal/mol with α-glucosidase, both of which are at relatively low levels, suggesting that ACB can form stable complexes with both target enzymes. In terms of binding mode, the hydrogen-bonding interactions of acarbose with the α-amylase receptor involved five residues, namely GLU240, ALA307, GLU233, ASP197, and HIS305, while hydrophobic interactions were mediated by TYR151, and the van der Waals contact network encompassed 11 residues including ASN152, GLY238, ASP236, ILE235, LYS200, GLY306, LEU162, ALA198, TYR62, ARG195, and ASP300 (Figure 3b). For the α-glucosidase receptor, ACB formed a more extensive hydrogen-bonding network involving seven residues, namely ASP571, ASP443, ASP327, ASP203, THR205, ASP474, and ASN207, with hydrophobic interactions provided by PHE575, and the van der Waals contact surface included 13 residues: ARG202, THR204, LYS480, ARG598, GLY541, ASP542, HIS600, TRP441, ILE364, TYR299, MET444, ARG526, and SER448 (Figure 3d). Taken together, these results indicate that ACB exhibits stable binding conformations in both enzymes.

Dipyridamole formed stable complexes with both α-amylase (−6.5 kcal/mol) and α-glucosidase (−6.0 kcal/mol), with binding energies close to those of ACB (−6.7 and −6.6 kcal/mol). In terms of binding mode, dipyridamole interacted with α-amylase primarily through hydrogen bonds involving ASP197 and ASP300, supplemented by hydrophobic and van der Waals contacts. Its binding to α-glucosidase involved a more intricate interaction network, including four hydrogen-bonding residues, one Pi-Anion interaction with ASP203, and one carbon–hydrogen bond with THR205, which may compensate for the relatively limited number of hydrogen bonds. Several key residues, such as ARG202, THR204, and TYR299, were shared between the two enzymes, suggesting they represent common binding hotspots. Overall, dipyridamole displayed dual-target inhibitory potential, with a more diverse binding profile toward α-glucosidase, offering insights for future structural optimization. Although molecular docking predictions indicate that dipyridamole can form thermodynamically stable complexes with the target enzymes, the actual in vivo efficacy is subject to multiple complex factors, including absorption, distribution, metabolism, excretion, target accessibility, and compensatory regulation, which cannot be fully captured by computational simulations. Therefore, the docking results above provide only an approximate estimate of binding affinity and should therefore be regarded as preliminary theoretical evidence. Further in vivo glucose tolerance tests in animal models are warranted to objectively assess the actual enzyme-inhibitory activity of dipyridamole and its potential for further development.

### 3.4. Molecular Dynamics Simulation Results

**Figure 4:**
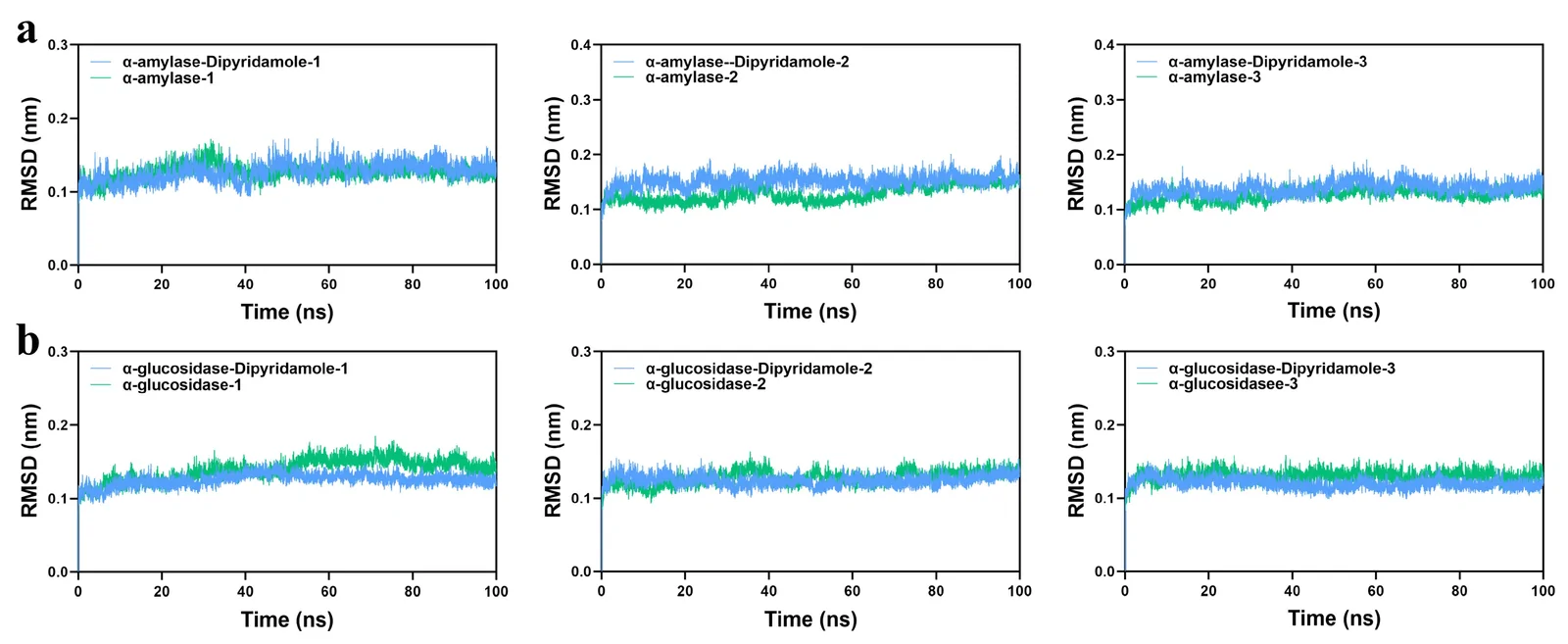
RMSD values of the protein–ligand complexes over time: (**a**) α-amylase; (**b**) α-glucosidase.

RMSD is a reliable indicator of the conformational stability of proteins and ligands, as well as a measure of the deviation of atomic positions from their initial coordinates [38]. The smaller the deviation, the better the conformational stability. The results of the three independent molecular dynamics simulations are presented in Figure 4. For the α-amylase system, both the apo protein and the α-amylase-dipyridamole complex underwent rapid relaxation during the early stage of the simulations and entered a stable plateau phase after approximately 20 ns, with mean RMSD values of 0.13 ± 0.01 nm and 0.14 ± 0.01 nm, respectively (Figure 4a). The overall RMSD levels were comparable between the bound and unbound states, indicating that dipyridamole binding did not appreciably perturb the global backbone conformation of α-amylase. For the α-glucosidase system, both the complex and the apo protein approached equilibrium after approximately 10 ns, with mean RMSD values of 0.12 ± 0.01 nm and 0.13 ± 0.01 nm, respectively (Figure 4b). Comparison between the apo and complex forms again revealed no large-scale backbone rearrangement induced by ligand binding. Taken together, under the present 100 ns simulation conditions, both the apo proteins and the dipyridamole complexes of α-amylase and α-glucosidase achieved satisfactory dynamical convergence, with the ligand remaining stably accommodated within the respective active sites.

**Figure 5:**
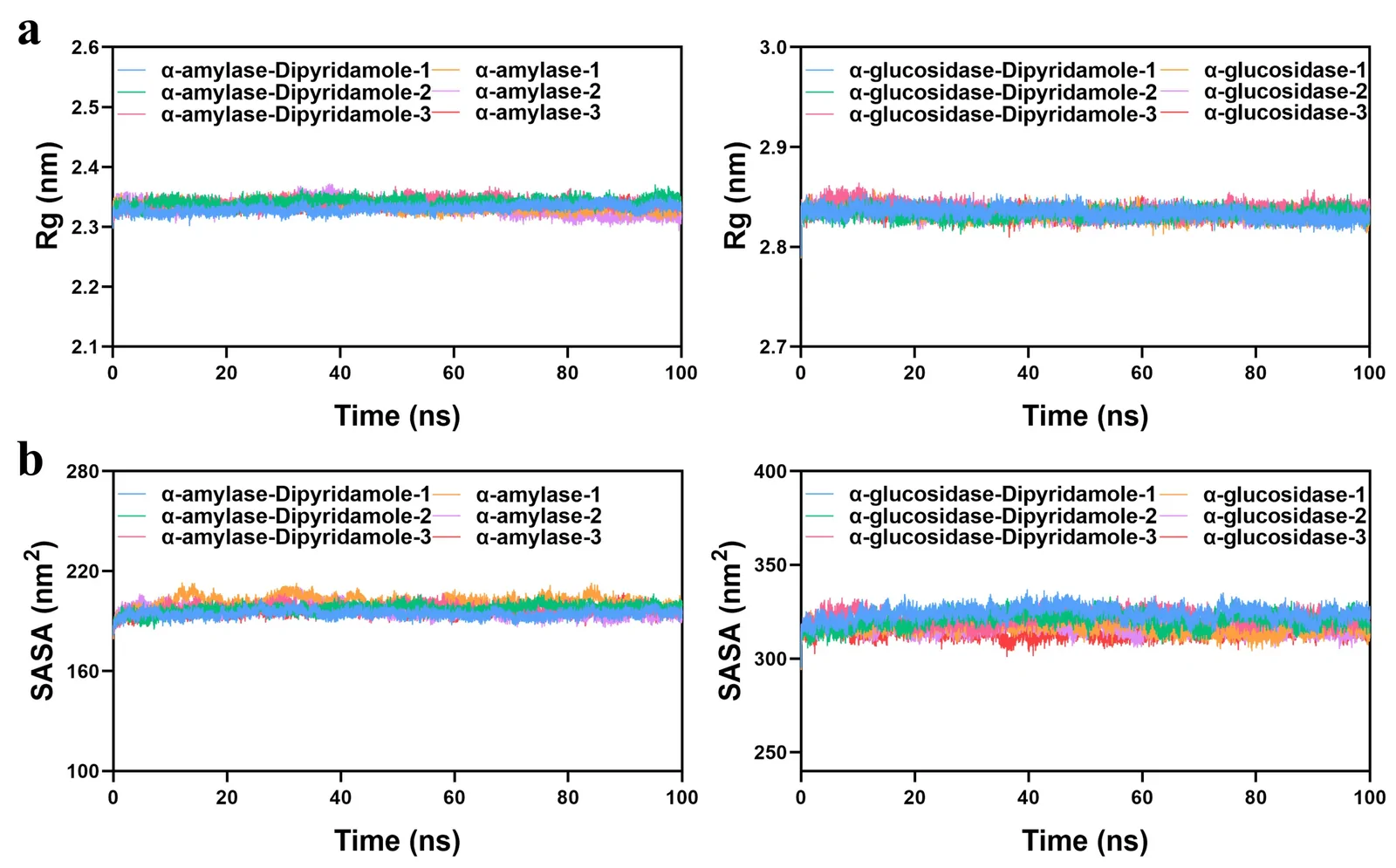
(**a**) Rg values of the protein–ligand complexes over time; (**b**) SASA values of the protein–ligand complexes over time.

Further analysis revealed that the radius of gyration (Rg) and solvent-accessible surface area (SASA) of the apo proteins and their corresponding complexes (α-amylase–dipyridamole and α-glucosidase–dipyridamole) remained relatively stable throughout the simulation trajectories (Figure 5). These observations suggest that binding of the small molecule did not induce appreciable expansion or contraction of the target proteins during the course of conformational dynamics.

**Figure 6:**
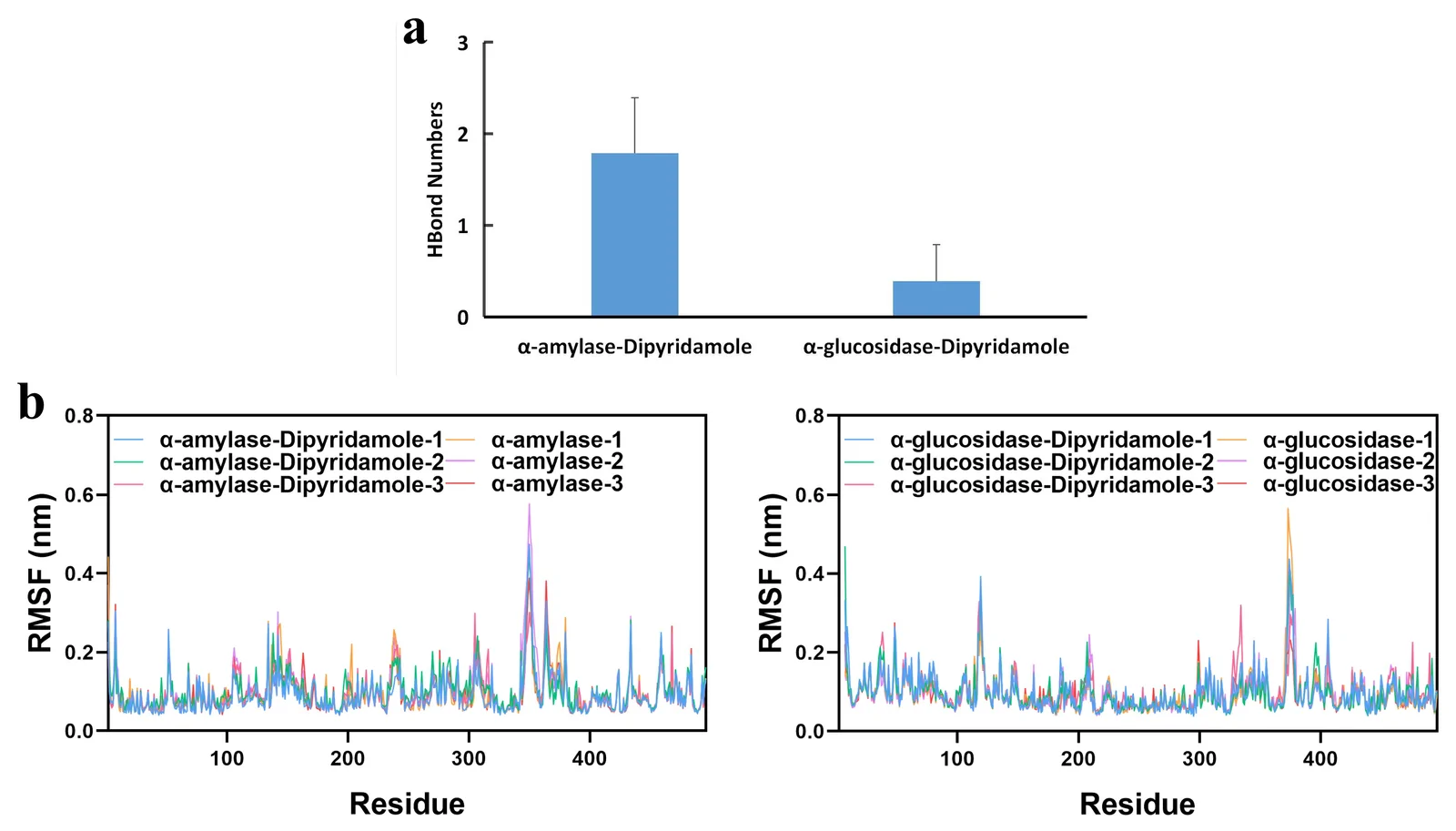
(**a**) Number of hydrogen bonds (H Bonds) in the protein–ligand complexes over time; (**b**) RMSF values of the protein–ligand complexes.

Hydrogen bonds play a critical role in mediating protein–ligand interactions. The number of hydrogen bonds formed between the small molecule and the target protein over the simulation trajectories is presented in Figure 6a. In both the α-amylase–dipyridamole and α-glucosidase–dipyridamole systems, a persistent population of protein–ligand hydrogen bonds were maintained throughout the simulations. Notably, the α-amylase–dipyridamole complex exhibited a consistently higher hydrogen bond count, suggesting the establishment of a more extensive hydrogen-bonding network in this system.

RMSF reflects the flexibility of amino acid residues in a protein [39]. As shown in Figure 6b, the RMSF profiles of the apo proteins and their corresponding complexes (α-amylase–dipyridamole and α-glucosidase–dipyridamole) exhibited a high degree of overlap. The RMSF values for the majority of domain residues remained within the range of 0–0.30 nm, indicating that the folded regions of the protein are generally rigid. Comparison of the trajectories between the apo proteins and the complexes revealed that no obvious flexibility fluctuations (either increase or decrease) occurred in the active-site residues of either protein upon ligand binding, suggesting that dipyridamole does not markedly affect the local dynamic behavior of the residues in α-amylase and α-glucosidase.

**Figure 7:**
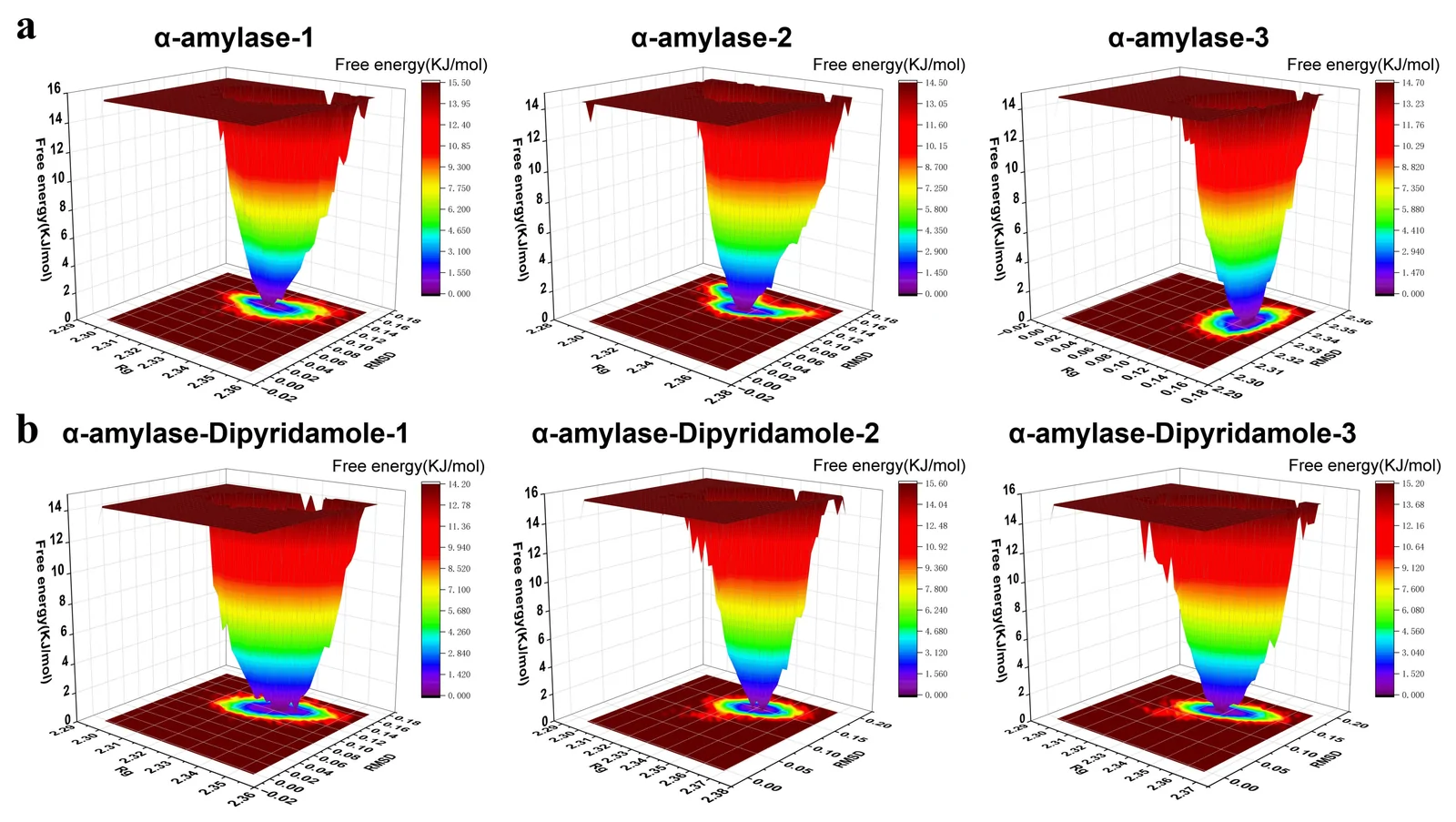
Free energy landscapes of (**a**) apo α-amylase and the (**b**) α-amylase–dipyridamole complex.

**Figure 8:**
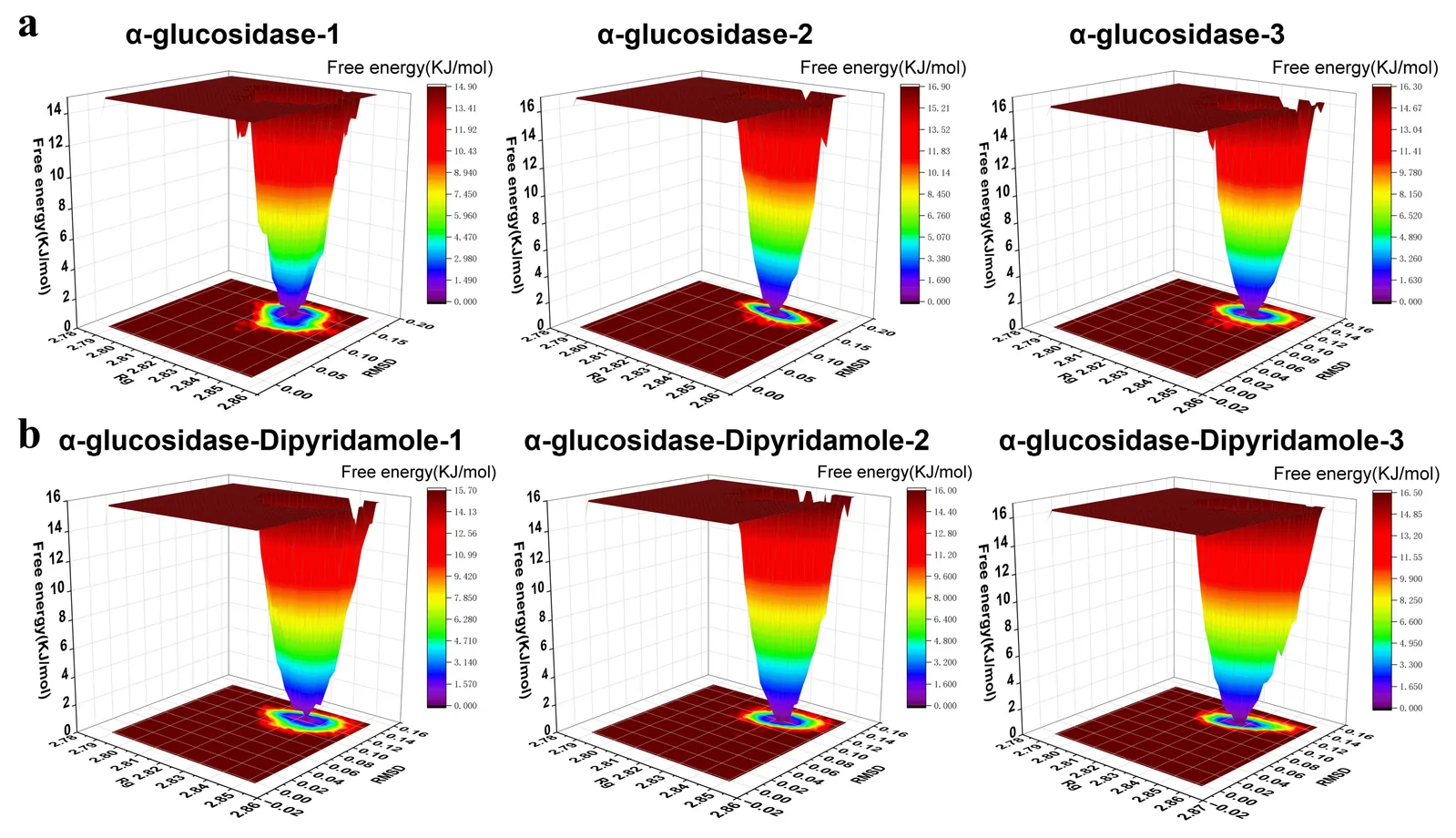
Free energy landscapes of (**a**) apo α-glucosidase and the (**b**) α-glucosidase–dipyridamole complex.

The free energy landscapes (FEL) of the systems are presented in Figure 7 and Figure 8, with color mapping indicating relative free energy values—red regions correspond to higher free energy, whereas blue regions denote lower free energy. It can be observed that both the apo proteins (α-amylase and α-glucosidase) and their corresponding dipyridamole complexes (α-amylase–dipyridamole and α-glucosidase–dipyridamole) each exhibited a single, compact low-energy conformational basin, characterized by a deep and well-localized energy well. Comparison between the apo and bound states revealed no additional discrete low-energy basins upon ligand binding, and the overall topographic features of the free energy landscapes remained highly similar between the two forms.

**Figure 9:**
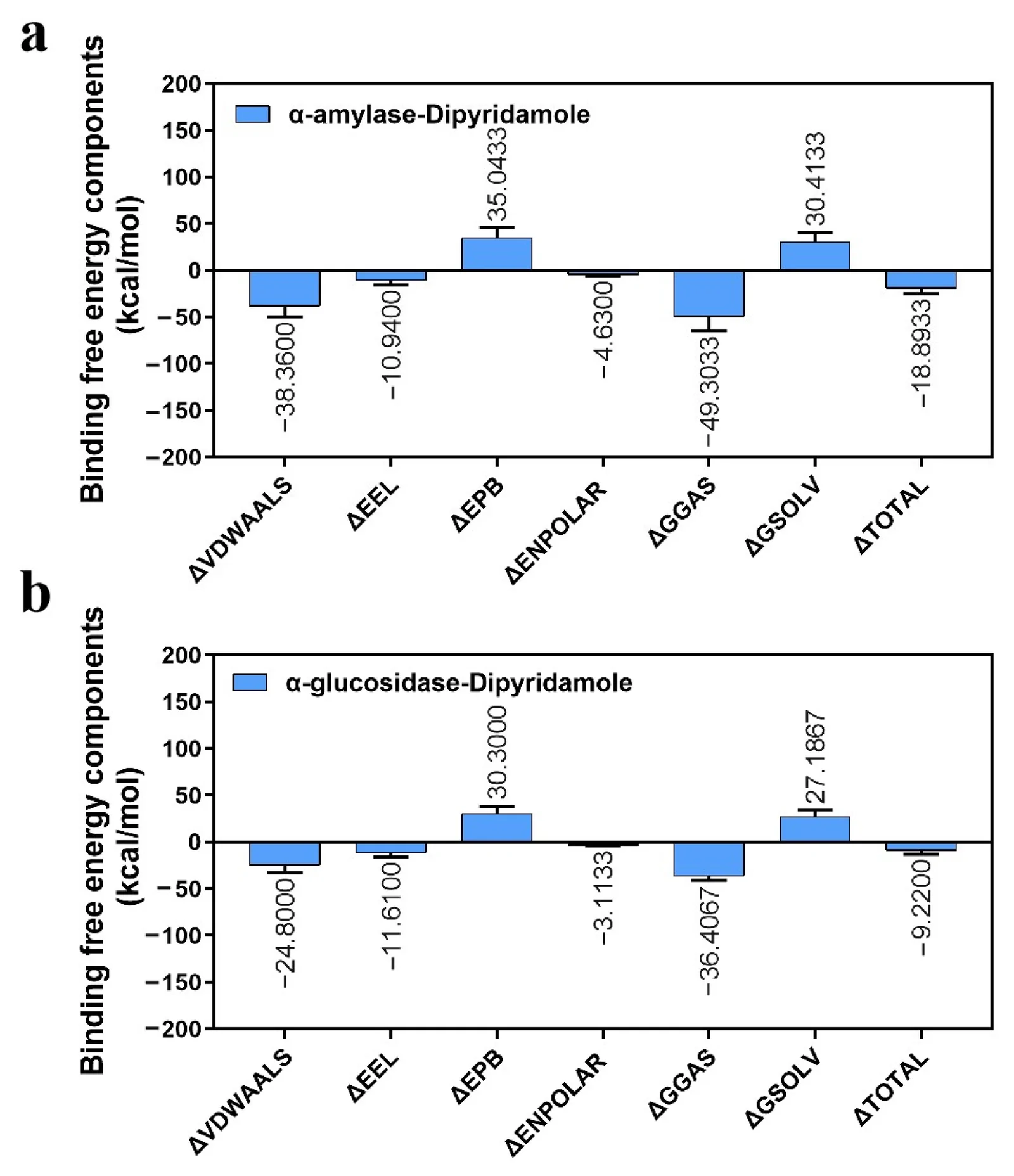
MM-PBSA binding free energy analysis of the protein–ligand complexes, (**a**) α-amylase–dipyridamole complex; (**b**) α-glucosidase–dipyridamole complex.

Subsequently, the binding free energies between the small molecule and the target proteins were estimated using the MM/PBSA method based on the equilibrated complex conformations (Figure 9). The calculated binding free energies for the α-amylase–dipyridamole and α-glucosidase–dipyridamole complexes were −18.89 ± 5.83 kcal/mol and −9.22 ± 3.95 kcal/mol, respectively. The negative values indicate favorable binding affinity of dipyridamole toward both target proteins, with more negative values corresponding to stronger binding. Among the two systems, the α-amylase–dipyridamole complex yielded the lowest MM/PBSA value, suggesting that this complex may possess relatively higher stability under the current simulation conditions.

In summary, both the apo proteins (α-amylase and α-glucosidase) and their respective dipyridamole complexes (α-amylase–dipyridamole and α-glucosidase–dipyridamole) exhibited relatively stable dynamical behavior under the current simulation conditions. Among them, the α-amylase–dipyridamole complex yielded a lower MM-PBSA binding free energy and maintained favorable hydrogen-bonding interactions. These observations suggest that dipyridamole may bind more favorably to α-amylase than to α-glucosidase. It should be noted that MM/PBSA calculations are, in essence, approximate evaluations of binding affinity rather than definitive measurements. Moreover, the present molecular dynamics results reflect conformational features only within the simulated timescale and should not be directly equated with true binding behavior in vivo. Therefore, the conclusions drawn from these computational approaches should be regarded as preliminary theoretical evidence, providing useful guidance for subsequent studies but not serving as conclusive proof of inhibitory potency. To objectively assess the genuine enzyme inhibitory activity of dipyridamole and its potential for further development, subsequent in vivo experiments in appropriate animal models are indispensable.

## 4. Conclusions

In this study, the antioxidant activity of dipyridamole and its interactions with α-amylase and α-glucosidase were preliminarily investigated through a combination of in vitro activity assays, molecular docking, and molecular dynamics simulations. Dipyridamole exhibited scavenging activity against ABTS+·, ·OH, and DPPH· radicals in vitro, and also showed certain inhibitory effects on both α-amylase and α-glucosidase. Mechanistic exploration revealed that the interactions are primarily mediated by the formation of non-covalent complexes. Molecular dynamics simulations further demonstrated that both protein–ligand complexes maintained structural convergence over the 100 ns simulation timescale. Nevertheless, the in vivo efficacy of dipyridamole remains to be substantiated through further investigations. Collectively, these findings suggest that this molecule may serve as a promising lead compound for further drug development and functional exploration.

Looking forward, further investigations are warranted to evaluate the antioxidant capacity of dipyridamole at the cellular level and to conduct in vivo pharmacodynamic studies, thereby validating its actual biological activity. In parallel, structure-based optimization of dipyridamole may be pursued according to the current binding conformations, with the aim of enhancing its affinity and selectivity toward the target proteins. Such efforts would provide more experimental evidence and lay the foundation for the expanded therapeutic applications of dipyridamole.

## Data Availability

The data that support the findings of this study are available from the corresponding author upon reasonable request.

## Abbreviations

The following abbreviations are used in this manuscript:

IDF	International Diabetes Federation
T2DM	Type 2 Diabetes Mellitus
ROS	Reactive Oxygen Species
RNS	Reactive Nitrogen Species
cAMP	Cyclic Adenosine Monophosphate
Vc	L-ascorbic acid
DPPH	2,2-Diphenyl-1-Picrylhydrazyl
ABTS	2,2′-Azinobis-(3-Ethylbenzthiazoline-6-Sulphonic acid)
ACB	Acarbose
RMSD	Root-Mean-Square Deviation
RMSF	Root-Mean-Square Fluctuation
Rg	Gyration Radius
SASA	Solvent-Accessible Surface Area

## References

[1] Genitsaridi I., Salpea P., Salim A., Sajjadi S.F., Tomic D., James S., Thirunavukkarasu S., Issaka A., Chen L., Basit A. (2026). 11th edition of the idf diabetes atlas: Global, regional, and national diabetes prevalence estimates for 2024 and projections for 2050. Lancet Diabetes Endocrinol..

[2] Zhang L., Li Z., Kong H., Ban X., Gu Z., Hong Y., Cheng L., Li C. (2024). Advances in microbial exopolysaccharides as α-amylase inhibitors: Effects, structure-activity relationships, and anti-diabetic effects in vivo. Int. J. Biol. Macromol..

[3] Zhao S., Cai S., Ding L., Yi J., Zhou L., Liu Z., Chu C. (2024). Exploring the blood glucose-lowering potential of the umami peptides ladw and eeaegt derived from tuna skeletal myosin: Perspectives from α-glucosidase inhibition and starch interaction. Foods.

[4] Gonzalez P., Lozano P., Ros G., Solano F. (2023). Hyperglycemia and oxidative stress: An integral, updated and critical overview of their metabolic interconnections. Int. J. Mol. Sci..

[5] Zhu Y., Wang K., Jia X., Fu C., Yu H., Wang Y. (2024). Antioxidant peptides, the guardian of life from oxidative stress. Med. Res. Rev..

[6] Balakumar P., Nyo Y.H., Renushia R., Raaginey D., Oh A.N., Varatharajan R., Dhanaraj S.A. (2014). Classical and pleiotropic actions of dipyridamole: Not enough light to illuminate the dark tunnel?. Pharmacol. Res..

[7] Chakrabarti S., Blair P., Wu C., Freedman J.E. (2007). Redox state of dipyridamole is a critical determinant for its beneficial antioxidant and antiinflammatory effects. J. Cardiovasc. Pharmacol..

[8] Li H., Horke S., Förstermann U. (2014). Vascular oxidative stress, nitric oxide and atherosclerosis. Atherosclerosis.

[9] Esmaeili S., Azizian S., Shahmoradi B., Moradi S., Shahlaei M., Khodarahmi R. (2019). Dipyridamole inhibits α-amylase/α-glucosidase at sub-micromolar concentrations; In-vitro, in-vivo and theoretical studies. Bioorganic Chem..

[10] Elsayed N., Marrez D.A., Ali M.A., El-Maksoud A.A.A., Cheng W., Abedelmaksoud T.G. (2022). Phenolic profiling and in-vitro bioactivities of corn (*Zea mays* L.) Tassel extracts by combining enzyme-assisted extraction. Foods.

[11] Liu H., Zhao C., Pang S., Shu Y., Chen L., Wang J., Bai H. (2026). Ultrasound-Assisted Extraction of Polyphenols from *Hericium erinaceus*: Optimization, Bioactivities and LC-MS-Based Chemical Profiling. Molecules.

[12] Kotora P., šeršeň F., Filo J., Loos D., Gregáň J., Gregáň F. (2016). The scavenging of dpph, galvinoxyl and abts radicals by imine analogs of resveratrol. Molecules.

[13] Padilla-Camberos E., Lazcano-Díaz E., Flores-Fernandez J.M., Owolabi M.S., Allen K., Villanueva-Rodríguez S. (2014). Evaluation of the inhibition of carbohydrate hydrolyzing enzymes, the antioxidant activity, and the polyphenolic content of *Citrus limetta* peel extract. Sci. World J..

[14] Benchchem Technical Support Center (2026). Minimizing the Impact of DMSO on Enzyme Activity in Biochemical Assays.

[15] Alam M.Z., Ramachandran T., Antony A., Hamed F., Ayyash M., Kamal-Eldin A. (2022). Melanin Is a Plenteous Bioactive Phenolic Compound in Date Fruits (*Phoenix dactylifera* L.). Sci. Rep..

[16] Yao M., Liu J., Zhou F., Li H., Wang R., Han Z., Liu J., Chen W., Liu G., Yang S. (2025). A-glucosidase inhibitory activity of polyphenol-rich sugarcane extract: Screening and mechanistic insights based on biolayer interferometry-mass spectrometry. Front. Nutr..

[17] Berman H.M., Westbrook J., Feng Z., Gilliland G., Bhat T.N., Weissig H., Shindyalov I.N., Bourne P.E. (2000). The Protein Data Bank. Nucleic Acids Res..

[18] Morris G.M., Huey R., Lindstrom W., Sanner M.F., Belew R.K., Goodsell D.S., Olson A.J. (2009). AutoDock4 and AutoDockTools4: Automated docking with selective receptor flexibility. J. Comput. Chem..

[19] Trott O., Olson A.J. (2010). AutoDock Vina: Improving the speed and accuracy of docking with a new scoring function, efficient optimization, and multithreading. J. Comput. Chem..

[20] Abraham M.J., Murtola T., Schulz R., Páll S., Smith J.C., Hess B., Lindahl E. (2015). GROMACS: High Performance Molecular Simulations through Multi-Level Parallelism from Laptops to Supercomputers. SoftwareX.

[21] Lindorff-Larsen K., Piana S., Palmo K., Maragakis P., Klepeis J.L., Dror R.O., Shaw D.E. (2010). Improved Side-Chain Torsion Potentials for the Amber ff99SB Protein Force Field. Proteins.

[22] Wang J., Wolf R.M., Caldwell J.W., Kollman P.A., Case D.A. (2004). Development and Testing of a General Amber Force Field. J. Comput. Chem..

[23] Chen X., Xie N., Feng L., Huang Y., Wu Y., Zhu H., Tang J., Zhang Y. (2025). Oxidative stress in diabetes mellitus and its complications: From pathophysiology to therapeutic strategies. Chin. Med. J..

[24] Bibi Sadeer N., Montesano D., Albrizio S., Zengin G., Mahomoodally M.F. (2020). The Versatility of Antioxidant Assays in Food Science and Safety-Chemistry, Applications, Strengths, and Limitations. Antioxidants.

[25] Martinelli E., Granato D., Azevedo L., Gonçalves J.E., Lorenzo J.M., Munekata P.E.S., Simal-Gandara J., Barba F.J., Carrillo C., Rajoka M.S.R. (2021). Current Perspectives in Cell-Based Approaches towards the Definition of the Antioxidant Activity in Food. Trends Food Sci. Technol..

[26] Sharon J.P., Beatrice D.A., Priyadarshini R.D., Haokip T. (2025). Bioactive potential of non-fermented and fermented Kullakar rice porridge:in vitro evaluation on the therapeutic properties. Prospects Pharm. Sci..

[27] Parhizkar E., Rashedinia M., Karimi M., Alipour S. (2018). Design and development of vitamin c-encapsulated proliposome with improved in-vitro and ex-vivo antioxidant efficacy. J. Microencapsul..

[28] Idamokoro E.M., Falowo A.B., Oyeagu C.E., Afolayan A.J. (2020). Multifunctional activity of vitamin e in animal and animal products: A review. Anim. Sci. J..

[29] Riyaphan J., Pham D., Leong M.K., Weng C. (2021). In silico approaches to identify polyphenol compounds as α-glucosidase and α-amylase inhibitors against type-ii diabetes. Biomolecules.

[30] Deng Y., Lin-Shiau S., Shyur L., Lin J. (2015). Pu-erh tea polysaccharides decrease blood sugar by inhibition of α-glucosidase activity in vitro and in mice. Food Funct..

[31] Liu Z., Yang Y., Dong W., Liu Q., Wang R., Pang J., Xia X., Zhu X., Liu S., Shen Z. (2019). Investigation on the Enzymatic Profile of Mulberry Alkaloids by Enzymatic Study and Molecular Docking. Molecules.

[32] Guan S., Han X., Li Z., Xu X., Cui Y., Chen Z., Zhang S., Chen S., Shan Y., Wang S. (2022). Exploration of the Interactions between Maltase–Glucoamylase and Its Potential Peptide Inhibitors by Molecular Dynamics Simulation. Catalysts.

[33] Bharathkumar H., Sundaram M.S., Jagadish S., Paricharak S., Hemshekhar M., Mason D., Kemparaju K., Girish K.S., Basappa B.A., Rangappa K.S. (2014). Novel benzoxazine-based aglycones block glucose uptake in vivo by inhibiting glycosidases. PLoS ONE.

[34] Moussa A.Y., Alanzi A., Luo J., Chung S.K., Xu B. (2024). Potential anti-obesity effect of saponin metabolites from adzuki beans: A computational approach. Food Sci. Nutr..

[35] Inohara-Ochiai M., Nakayama T., Goto R., Nakao M., Ueda T., Shibano Y. (1997). Altering substrate specificity of *Bacillus* sp. SAM1606 alpha-glucosidase by comparative site-specific mutagenesis. J. Biol. Chem..

[36] Kashiwabara S., Azuma S., Tsuduki M., Suzuki Y. (2000). The Primary Structure of the Subunit in *Bacillus thermoamyloliquefaciens* KP1071 Molecular Weight 540,000 Homohexameric Alpha-Glucosidase II Belonging to the Glycosyl Hydrolase Family 31. Biosci. Biotechnol. Biochem..

[37] Zhou H., Xiong Z., Ma X., Dai L., Kuang L., Deng R., Lv X., Tuo X. (2023). A multispectral study and computer simulation on the interaction of pomalidomide with human serum albumin. J. Mol. Liq..

[38] Sargsyan K., Grauffel C., Lim C. (2017). How molecular size impacts rmsd applications in molecular dynamics simulations. J. Chem. Theory Comput..

[39] Broz M., Oostenbrink C., Bren U. (2024). The effect of microwaves on protein structure: Molecular dynamics approach. J. Chem. Inf. Model..

